# Draft genome sequence for virulent and avirulent strains of *Xanthomonas arboricola* isolated from *Prunus* spp. in Spain

**DOI:** 10.1186/s40793-016-0132-3

**Published:** 2016-01-28

**Authors:** Jerson Garita-Cambronero, Ana Palacio-Bielsa, María M. López, Jaime Cubero

**Affiliations:** Instituto Nacional de Investigación y Tecnología Agraria y Alimentaria, Madrid, Spain; Centro de Investigación y Tecnología Agroalimentaria de Aragón, Zaragoza, Spain; Instituto Valenciano de Investigaciones Agrarias, Valencia, Spain

**Keywords:** *Xanthomonas arboricola*, *Prunus* spp., Stone fruits, Bacterial spot disease, Plant pathogenic bacteria

## Abstract

*Xanthomonas arboricola* is a species in genus *Xanthomonas* which is mainly comprised of plant pathogens. Among the members of this taxon, *X. arboricola* pv. pruni, the causal agent of bacterial spot disease of stone fruits and almond, is distributed worldwide although it is considered a quarantine pathogen in the European Union. Herein, we report the draft genome sequence, the classification, the annotation and the sequence analyses of a virulent strain, IVIA 2626.1, and an avirulent strain, CITA 44, of *X. arboricola* associated with *Prunus* spp. The draft genome sequence of IVIA 2626.1 consists of 5,027,671 bp, 4,720 protein coding genes and 50 RNA encoding genes. The draft genome sequence of strain CITA 44 consists of 4,760,482 bp, 4,250 protein coding genes and 56 RNA coding genes. Initial comparative analyses reveals differences in the presence of structural and regulatory components of the type IV pilus, the type III secretion system, the type III effectors as well as variations in the number of the type IV secretion systems. The genome sequence data for these strains will facilitate the development of molecular diagnostics protocols that differentiate virulent and avirulent strains. In addition, comparative genome analysis will provide insights into the plant-pathogen interaction during the bacterial spot disease process.

## Introduction

*Xanthomonas arboricola* [1] are plant associated bacteria in nine pathovars with a diverse range of biotic relationships [2, 3]. Within this taxon, plant pathogenic strains with non-pathogenic strains have been described. Bacterial spot of *Prunus* spp. (*X. arboricola* pv. pruni), bacterial blight of *Juglans* spp. (*X. arboricola* pv. juglandis) and *Corylus* spp. (*X. arboricola* pv. corylina) are among the most harmful diseases of these tree hosts. These bacterial diseases are distributed worldwide and the causal bacteria are regulated in several countries including the European Union, where *X. arboricola* pv. pruni is a quarantine pathogen [4, 5].

Within the pathovars, *X. arboricola* pv. pruni is a major threat to cultivated, exotic and ornamental *Prunus* species. This bacterium has been identified as a pathogen of *P. armeniaca**,**P. avium**,**P. buergeriana**,**P. cerasus**P. crassipes*, *P. davidiana*, *P. domestica*, *P**. donarium*, *P. dulcis*, *P. laurocesasus*, *P. mume*, *P. persica* and *P. salicina* [6]. During the last decade, some local outbreaks of bacterial spot in Spain have been reported on almond, peach, nectarine and plum [7]. For initial characterization of the bacterial strains isolated from Spanish outbreaks of bacterial spot, we performed a polyphasic study based on a multilocus sequence analysis, as well as some phenotypic characters [8]. After the characterization that showed the presence of different molecular and phenotypic variants, selected strains were analysed to assess the differences at the whole genome level.

Genome sequencing of *X. arboricola* strains has been completed for five strains isolated from walnut, three from peach, two from *Musa* sp., one from almond [9], one from barley [10] and one from Turkish hazel [11]. Genome sequencing includes the plasmid pXap41 [12], present in the *X. arboricola* pv. pruni strains. All these sequences have been deposited in the NCBI database. Four genome sequences are available for pathogenic strains from *Prunus*, identified as *X. arboricola* pv. pruni. However, with the exception of the strain CITA 33 isolated from almond (*P. amygdalus*, syn. *P. dulcis*) in Spain [9], no detailed information about features of those genomes have been published. In the same way, there are no sequenced strains isolated from Japanese plum (*P. salicina*) or cherry rootstock (*P. mahaleb*). In addition, no avirulent strain of *X. arboricola* from *Prunus* spp. has been analysed at the whole-genome level. The occurrence of avirulent strains is of particular importance for a quarantine pathogen like *X. arboricola* pv. pruni with respect to accurate diagnosis of virulent strains.

Herein we present draft genome sequences for two *X. arboricola* strains: an avirulent strain, CITA 44, isolated from *P. mahaleb,* and *X. arboricola* pv. pruni strain, IVIA 2626.1, isolated from *P. salicina* cv. Fortuna, which differs from other sequenced strains in phenotypical features and virulence on several hosts [9]. The genome analysis of these two strains as well as comparison with other related strains should provide insight into the genetics of the pathogenesis process in *X. arboricola* strains associated with the bacterial spot disease of stone fruits and almond.

## Organism information

### Classification and features

Strain CITA 44 was isolated in 2009 from asymptomatic leaves of Santa Lucía SL-64 cherry rootstock (*P. mahaleb*) in a nursery located in the north-eastern Spanish region of Aragón. This strain showed flagella associated swarming and swimming motility on 0.5 % agar PYM plates and 0.3 % agar MMA plates, respectively. Additionally, strain CITA 44 showed type IV pili associated twitching motility in the interstitial surface between 1 % agar PYM layer and the plastic plate surface. According to the atomized oil assay [13], this strain produced surfactant compounds on 1.5 % agar LB plates after 24 h at 27 °C. In accordance with a detached leaf assay, conducted with a cotton swap damped with 1 × 10^8^ CFU/ml, on almond cv. Ferraduel, apricot cv. Canino, peach cv. Calanda and European plum (*P. domestica*) cv. Golden Japan, *X. arboricola* strain CITA 44 did not cause bacterial spot symptoms at 28 days post inoculation (dpi). Despite this lack of symptoms, the bacterium could be re-isolated after such period.

*X. arboricola* pv. pruni strain IVIA 2626.1 was isolated from symptomatic leaves of Japanese plum (*P. salicina* cv. Fortune) in the southwestern Spanish region of Extremadura in 2002. This strain showed swarming, swimming and twitching type motility as well as production of surfactant compounds in the same culture conditions described above for strain CITA 44. In addition, according to the detached leaf assay described previously, strain IVIA 2626.1 was able to produce bacterial spot symptoms on almond, peach and European plum but not on apricot after 28 dpi.

Classification of the strains was performed using an MLSA approach based on the partial sequences of the housekeeping genes *atpD*, *dnaK*, *efP*, *fyuA*, *glnA*, *gyrB* and *rpoD* of the strains CITA 44 and IVIA 2626.1 as well as related strains of *X. arboricola* [3]. Nucleotide sequences were aligned with Clustal W and both ends of each alignment were trimmed (*atpD* 750 bp, *dnaK* 759 bp, *efP* 339 bp, *fyuA* 753 bp, *glnA* 675 bp, *gyrB* 735 bp and *rpoD* 756 bp) and concatenated to a total length sequence of 4,620 nucleotide positions. The phylogenetic tree was constructed using the maximum likelihood method implemented in MEGA 6.0 [14] using 1,000 bootstrap re-samplings. According to the phylogenetic analysis, strain CITA 44 belongs to the species *X. arboricola*, nevertheless, this strain could not be associated to any of the pathovars of this species. The concatenated sequence similarity among this strain and the other *X. arboricola* strains analysed varied from 97.08 % to 98.79 %. In contrast, strain IVIA 2626.1 was clustered in a group with the pathotype strain *X. arboricola* pv. pruni CFBP 2535, isolated from *P. salicina* in New Zealand, with a sequence similarity of 100 %.

**Fig. 1:**
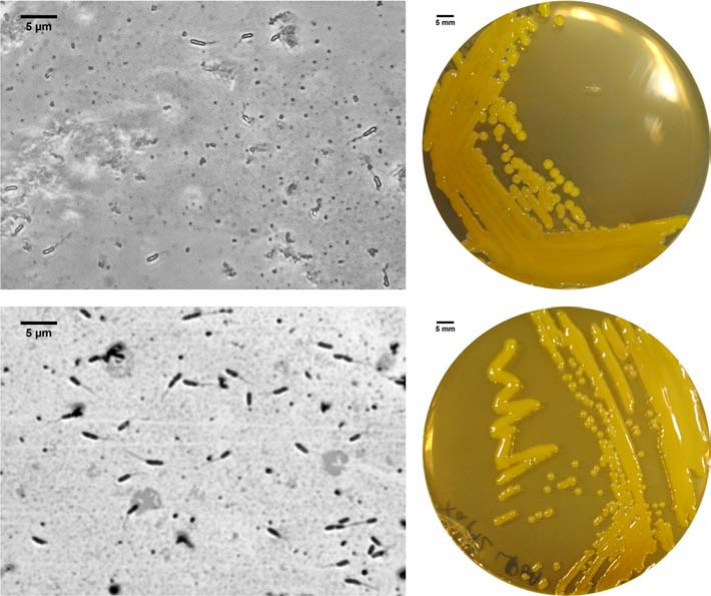
Images of *X. arboricola* CITA 44 (up) and *X. arboricola* pv. pruni IVIA 2626.1 (down) cells using contrast-phase microscopy (left) and the appearance of the colony morphology after 48 h growing on YPGA agar medium at 27 °C (right). Flagella was stained (left) as described previously [63]

*X. arboricola*CITA 44 and *X. arboricola* pv. pruni IVIA 2626.1 strains are Gram-negative, non-sporulating, rod-shaped, motile cells with a single polar flagellum. Rod-shaped cells of CITA 44 are approximately 0.6 μm in width and 1.4–2.5 μm in length. Rod-shaped cells of IVIA 2626.1 are approximately 0.7 μm in width and 1.7–2.5 μm in length. These strains formed 2.0–3.0 mm colonies within 48 h at 27 °C on YPGA 1.5 % agar plates [15]. Both strains formed mucoid, circular, yellow colonies with a convex elevation and an entire margin (Fig. 1). Strains CITA 44 and IVIA 2626.1 grew in the nutritive culture media PYM [16] and LB [17], as well as in the minimal medium A [18]. According to the Biolog GN2 system, both strains metabolized α-D-glucose, α-keto glutamic acid, bromosuccinic acid, D-cellobiose, D-fructuose, D-mannose, D-psicose, D-threalose, glycyl-L-glutamic acid, L-glutamic acid, L-serine, pyruvic acid methyl ester, succinic acid, succinic acid mono-methyl-ester, sucrose and Tween 40. The carbon compound D-saccharic acid was only utilized by strain CITA 44. Dextrin and L-proline were only metabolized by strain IVIA 2626.1. In addition to this analysis, strain CITA 44 hydrolysed casein and starch, while strain IVIA 2626.1 did not (Table 1).

**Table 1:** Classification and general features of two *Xanthomonas arboricola* strains according to the MIGS recommendation [19] published by the Genomic Standards Consortium [53]

MIGS ID	Property	Term	Evidence code^a^
	Classification	Domain: *Bacteria*	TAS [54]
		Phylum: *Proteobacteria*	TAS [55]
		Class: *Gammaproteobacteria*	TAS [56–58]
		Order: *Lysobacterales*	TAS [57, 59, 60]
		Family: *Lysobacteraceae*	TAS [57, 58, 60]
		Genus: *Xanthomonas*	TAS [1]
		Species: *Xanthomonas arboricola*	IDA
		Strain: CITA 44, IVIA 2626.1	IDA
	Gram stain	Negative	TAS [61]
	Cell shape	Rod-shaped	IDA
	Motility	Motile	IDA
	Sporulation	Non-sporulating	IDA
	Temperature range	4-37 °C	TAS [1]
	Optimum temperature	27 °C	IDA
	pH range; Optimum	7.5-8.5	TAS [61]
	Carbon source	α-D-glucose, α-keto glutaric acid, bromosuccinic acid, D-cellobiose, D-fructuose, D-mannose, D-psicose, D-saccharic acid (only strain CITA 44), D-threalose, Dextrin (only strain IVIA 2626.1), glycyl-L-glutamic acid, L-glutamic acid, L- proline (only strain IVIA 2626.1), L-serine, pyruvic acid methyl ester, succinic acid, succinic acid mono-methyl ester, Sucrose, tween 40	IDA
	Energy metabolism	Chemoorganotrophic	TAS [1]
MIGS-6	Habitat	Plants	IDA TAS [1]
MIGS-6.3	Salinity	0-6.0 % NaCl	TAS [1]
MIGS-10	Extrachromosomal elements	None in CITA 44, one in IVIA 2626.1	IDA, TAS [12]
MIGS-22	Oxygen requirement	Aerobic	IDA
MIGS-15	Biotic relationship	Epiphyte and endophyte	TAS [1]
MIGS-14	Pathogenicity	CITA 44 is avirulent; IVIA 2626.1 is virulent on almond, peach and European plum	IDA
	Host	Mahaleb cherry (*P. mahaleb*) (CITA 44) and plum (*P. salicina*) (IVIA 2626.1)	IDA
	Host taxa ID	129217 (CITA 44) and 88123 (IVIA 2626.1)	
	Isolation source	Leaf	IDA
MIGS-4	Geographic location	Spain	IDA
MIGS-5	Sample collection	2002 (IVIA 2626.1) and 2009 (CITA 44)	IDA
MIGS-4.1	Latitude	Unknown	NAS
MIGS-4.2	Longitude	Unknown	NAS
MIGS-4.4	Altitude	Unknown	NAS

^a^Evidence codes - IDA: Inferred from Direct Assay; TAS: Traceable Author Statement (i.e., a direct report exists in the literature); NAS: Non-traceable Author Statement (i.e., not directly observed for the living, isolated sample, but based on a generally accepted property for the species, or anecdotal evidence). These evidence codes are from the Gene Ontology project [62]

**Fig. 2:**
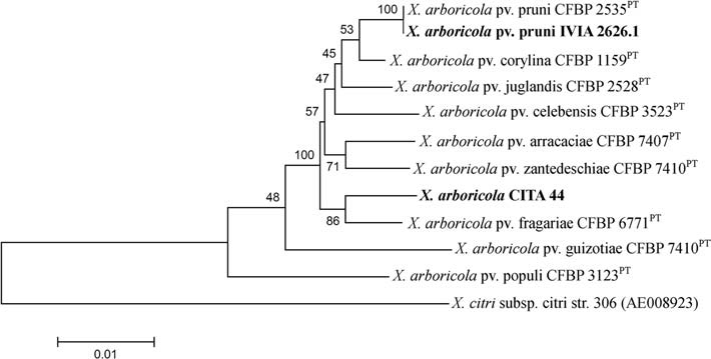
Phylogenetic tree highlighting the position of two *X. arboricola* strains (shown in bold) relative to the pathotype strains (PT) of *X. arboricola. X. citri* subsp. *citri* str. 306 [64, 65] was used as an outgroup. The tree was built based on the comparison of concatenated nucleotide sequences of seven housekeeping genes (*atpD*, *dnaK*, *efP*, *fyuA*, *glnA*, *gyrB* and *rpoD*) [3]. Sequences were first aligned and concatenated. The phylogenetic tree was constructed by using MEGA 6.0 software [13] with Maximum Likelihood method based on Tamura-Nei model. Bootstrap values (1,000 replicates) are shown at the branch points. GenBank accession number of *X. citri* subsp. *citri* str. 306 genome sequence is shown in parenthesis; accession numbers associated to the housekeeping loci of the pathotype strains can be found in a previous study [3]

Minimum information about genome sequence [19] of *X. arboricola* strain CITA 44 and *X. arboricola* pv. pruni strain IVIA 2626.1, as well as their phylogenetic position, are provided in Table 1 and Fig. 2.

## Genome sequencing information

### Genome project history

*X. arboricola* strain CITA 44 and *X. arboricola* pv. pruni strain IVIA 2626.1 were selected for comparative whole sequencing analysis as *X. arboricola* strains isolated from *Prunus* spp. with several different phenotypic characters including virulence. Comparative genomics among the avirulent strain CITA 44 and the available *Prunus-*pathogenic strains including IVIA 2626.1 should be useful for identifying the molecular determinants associated with pathogenesis as well as those associated with host resistance and for diagnostic characterization of *X. arboricola* strains causing bacterial spot of *Prunus* spp. Whole Genome Shotgun Projects have been deposited at DDBJ/EMBL/GenBank under the accession numbers LJGM00000000 and LJGN00000000. The versions described in this paper are versions LJGM01000000 and LJGN01000000. Table 2 summarizes the project information and its association with MIGS.

**Table 2:** Project information

MIGS ID	Property	Term/Strains	
		CITA 44	IVIA 2626.1
MIGS 31	Finishing quality	Draft	Draft
MIGS 28	Libraries used	One 400 bp Ion Torrent library	One 400 bp Ion Torrent library
MIGS 29	Sequencing platforms	Ion Torrent PGM	Ion Torrent PGM
MIGS 31.2	Fold coverage	198×	92×
MIGS 30	Assemblers	MIRA 4.0	MIRA 4.0
MIGS 32	Gene calling method	Glimmer 3.0 that used in the RAST pipeline	Glimmer 3.0 that used in the RAST pipeline
	Locus Tag	AN651	AN652
	Genbank ID	LJGM00000000	LJGN00000000
	GenBank Date of Release	06-October-2015	06-October-2015
	GOLD ID	Gp0124696	Gp0124697
	BIOPROJECT	PRJNA294649	PRJNA294655
MIGS 13	Source Material Identifier	CITA 44	IVIA 2626.1
	Project relevance	Agricultural, Environmental, Biotechnology, Plant-Bacteria Interaction	Agricultural, Environmental, Biotechnology, Plant-Bacteria Interaction

### Growth conditions and genomic DNA preparation

*X. arboricola* strain CITA 44 and *X. arboricola* pv. pruni strain IVIA 2626.1 are deposited and available at the bacterial collections of the Instituto Valenciano de Investigaciones Agrarias (IVIA, Valencia, Spain) and the Centro de Investigación y Tecnología Agroalimentaria de Aragón (CITA, Zaragoza, Spain). Both strains were streaked on 1.5 % agar LB plates and were grown for 48 h at 27 °C. A single colony of each strain was inoculated separately in 30 ml of LB broth and grown on an orbital shaker for 24 h at 27 °C. DNA from pure bacterial cultures was extracted using a QIAamp DNA miniKit (Qiagen, Barcelona, Spain) according to the manufacturer instructions. DNA quality and quantity were determined by 1 % agarose gel electrophoresis, as well as using the Qubit flurometer (Invitrogen) according to the Quant-it dsDNA BR Assay Kit (Invitrogen) manufacturer instructions, and by a spectrophotometry (NanoDrop 2000 spectrophotometer, Thermo Scientific). A 2.0 μg/μl aliquot of 200 ng/μl sample was submitted for the sequencing.

### Genome sequencing and assembly

The draft genome sequences for strains CITA 44 and IVIA 2626.1 were generated at the STAB VIDA Next Generation Sequencing Laboratory (Caparica, Portugal) using the Ion Torrent sequencing technology. Draft genome assembly of strain CITA 44 was based on 3,060,638 usable reads with a total base number of 948,933,067. The mean read length was 361.70 ± 93.50 and the mode read length was 385 bp. The draft genome assembly of IVIA 2626.1 was based on 2,317,319 reads, with a total base number of 461,361,072. The mean read length and the mode read length for this strain were 201.80 ± 85.30 bp and 241 bp, respectively. Genomic assemblies were constructed using MIRA 4.0 [20]. From the total of contigs generated, only those with a contig size above 500 bp and an average coverage above 99 in the case of CITA 44, and 40, in the case of IVIA 2626.1 were considered significant. Finally, 71 contigs (N50 = 120,981 bp; largest contig = 352,479 bp; average coverage = 198X) were generated for strain CITA 44 and for strain IVIA 2626.1, 214 contigs (N50 = 47,650; largest contig = 115,385; average coverage = 92X) were generated.

### Genome annotation

The assembled draft genome for both strains was annotated using the RAST platform and the gene-caller GLIMMER 3.02 [21, 22]. RNAmmer version 1.2 [23] and tRNAscan-SE version 1.21 [24] were used to predict rRNAS and tRNAS, respectively. Signal peptides and transmembrane domains were determined using the SignalP 4.1 server [25] and the TMHMM server version 2.0 [26], respectively. Assignment of genes to the COG database [27] and Pfam domains [28] was performed with the NCBI conserved domain database using an expected value threshold of 0.001 [29].

Major structural components associated with the flagellum [30, 31], the type IV pilus [32], the type III secretory system [33, 34] and the type III effectors [35, 36], as well as the type IV secretory system and effectors [37–39], were identified in the draft genome sequence for each strain. Initially, the query of those genes was based on the coding sequence regions automatically annotated by RAST, and were confirmed using the BLASTn and BLASTx tools available at NCBI. Those components which were not automatically annotated were found in the genome sequence using the progressive Mauve alignment method [40]. Nucleotide sequences of the genes used for these alignments were obtained from other xanthomonads in the NCBI gene database. Finally, the nucleotide sequence of the aligned regions was analysed using the BLAST approaches mentioned above. Those sequences with query coverage and identity percentage higher than 90 % were annotated. Additionally, the core components of the T3SS and T4SS were searched using the T346Hunter application [41]. T3Es and T4Es genes were predicted using the Effective database [42] after selection of the “gram-” parameter as organism type and the “plant set” parameter as classification module, and the SecReT4 tool [43], respectively. All the predicted genes were corroborated and annotated according to the BLAST parameters mentioned above.

## Genome properties

**Fig. 3:**
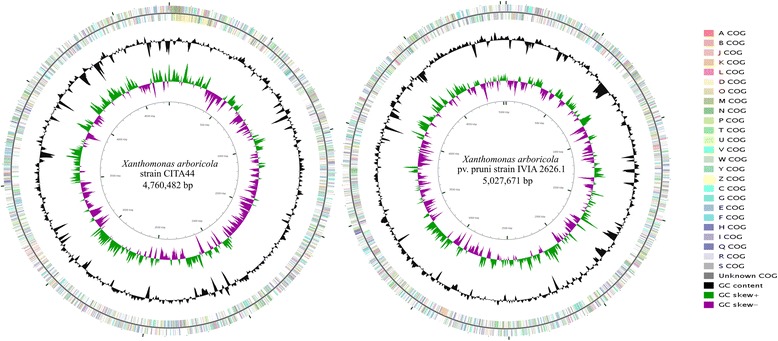
Graphical circular representation of the draft genome of *X. arboricola* CITA 44 and *X. arboricola* pv. pruni IVIA 2626.1. The contigs of both strains were ordered by Mauve [66] using the genome sequence of *X. campestris* pv. campestris ATCC 33913 [45, 46] as the reference. COG categories were assigned to genes by NCBI’s conserved domain database [29]. The circular map was constructed using CGView [67]. From outside to center: Genes on forward strand (colored by COG categories); genes on reverse strand (colored by COG categories); GC content; GC skew

The draft genome sequence of *X. arboricola* strain CITA 44 was 4,760,482 bp in length with an average GC content of 65.8 %, which is similar to that for other genomes of this species (65.4 to 66.0 %) reported in the NCBI genome database. For this strain, 4,306 genes were predicted and 4,250 were determined as protein coding genes. From these protein coding genes, 3,330 genes were assigned to a putative function and the remaining 920 were designated as hypothetical proteins. This strain presented 3 rRNA and 53 tRNA genes. In the case of the *X. arboricola* pv. pruni strain IVIA 2626.1, the draft genome sequence was 5,027,671 bp in length with an average GC content of 65.4 %, which is the same as for other strains of *X. arboricola* pv. pruni according to the NCBI database. A total of 4,770 genes were predicted and, among them, 4,720 were predicted as protein coding genes with 69.17 % assigned to a function and 30.83 % designated as hypothetical proteins. 50 RNA genes (3 rRNA and 47 tRNA genes) were predicted for this strain. The properties and characteristics associated with these genomes are presented in Table 3. The classification of the predicted protein coding genes into COG functional categories [44] is summarized in Fig. 3 and Table 4.

**Table 3:** Genome statistics

Attribute	Strain			
	CITA 44		IVIA 2626.1	
	Value	% of total	Value	% of total
Genome size (bp)	4,760,482	100.00	5,027,671	100.00
DNA coding (bp)	3,992,937	83.88	4,295,592	84.44
DNA G + C (bp)	3,134,520	65.80	3,288.794	65.40
Total genes	4306	100.00	4770	100.00
Protein coding genes	4250	98.62	4720	98.95
RNA genes	56	1.38	50	1.05
Pseudo genes	0	0.00	0	0.00
Genes with function prediction	3330	78.35	3265	69.17
Genes assigned to COGs	3137	73.81	3237	68.58
Genes with Pfam domains	3337	78.51	3433	72.73
Genes with signal peptides	526	12.37	545	11.55
Genes with transmembrane helices	1121	26.37	1221	25.86
CRISPR repeats	1	-	1	-

**Table 4:** Number of genes associated with general COG functional categories

Code	Strain				Description
	CITA 44		IVIA 2626.1		
	Value	% age	Value	% age	
J	218	5.13	217	6.70	Translation, ribosomal structure and biogenesis
A	1	0.02	2	0.06	RNA processing and modification
K	193	4.54	199	6.15	Transcription
L	111	2.61	127	3.92	Replication, recombination and repair
B	1	0.02	1	0.03	Chromatin structure and dynamics
D	35	0.82	40	1.23	Cell cycle control, cell division, chromosome partitioning
V	63	1.48	68	2.10	Defense mechanisms
T	211	4.96	208	6.42	Signal transduction mechanisms
M	225	5.29	235	7.26	Cell wall/membrane biogenesis
N	114	2.68	119	3.67	Cell motility
Z	2	0.05	2	0.06	Cytoskeleton
W	2	0.05	2	0.06	Extracellular structures
U	69	1.62	81	2.50	Intracellular trafficking and secretion
O	160	3.76	171	5.28	Posttranslational modification, protein turnover, chaperones
C	184	4.33	173	5.34	Energy production and conversion
G	220	5.18	215	6.64	Carbohydrate transport and metabolism
E	225	5.29	239	7.38	Amino acid transport and metabolism
F	76	1.79	76	2.35	Nucleotide transport and metabolism
H	153	3.60	144	4.45	Coenzyme transport and metabolism
I	156	3.67	156	4.82	Lipid transport and metabolism
P	218	5.13	212	6.55	Inorganic ion transport and metabolism
Q	57	1.34	63	1.95	Secondary metabolites biosynthesis, transport and catabolism
R	223	5.25	223	6.89	General function prediction only
S	213	5.01	223	6.89	Function unknown
X	7	0.16	41	1.27	Mobilome: prophages, transposons
-	1113	26.19	1483	31.42	Not in COGs

The total is based on the total number of protein coding genes in the annotated genome

## Insights from the genome sequence

Based on the phenotypic differences between CITA 44 and IVIA 2626.1 strains, selected genes associated with motility and pathogenicity were analysed (Table 5). No differences were observed for the structural components associated with bacterial flagella. A total of 30 out of the 31 components described for this organelle were identified [31], but neither of the two strains contained a homolog of the *flhE* gene. Regarding the 27 components associated with type IV pilus biogenesis and regulation in *Xanthomonas* [32, 45, 46], *fimX, pilD, pilE, pilL* and *pilW* genes were absent in strain CITA 44, whereas strain IVIA 2626.1 sequence did not contain homologs for *fimX* and *pilL* genes.

**Table 5:** Molecular components putatively involved in motility and pathogenesis

	Shared by CITA 44^a^ and IVIA 2626.1	Absent in CITA 44 and IVIA 2626.1	Unique in IVIA 2626.1
Flagella	*flgB, flgC, flgD, fLgE, flgF, flgG, flgH, flgJ, flgK, flgL, flgM, flgN, flhA, flhB, fliC, fliD, fliE, fliF, fliG, fliH, fliJ, fliK, fliL, fliM, fliN, fliO, fliP, fliQ, fliR, flK*	*flhE*	*-*
Type IV pilus	*fimT, pilA, pilB, pilC, pilF, pilG, pilI, pilJ, pilM, pilN, pilO, pilP, pilQ, pilR, pilS, pilT, pilU, pilV, pilX, pilY1, pilZ*	*fimX, pilL *	*pilD, pilE, pilW*
Type III Secretion System	*hrpG, hrpX*	*hpaF, hrpB5*	*hpa1, hpa2, hpaB, hpaF, hpaP, hrcC, hrcJ, hrcN, hrcQ, hrcR, hrcS, hrcT, hrcU, hrcV, hrpB1, hrpB2, hrpB4, hrpB5, hrpB7, hrpD5, hrpD6, hrpE, hrpF*
Type III effectors	*-*	*avrBs1, avrBs3, xopAA, xopAB, xopAC, xopAD, xopAE, xopAG, xopAJ, xopAK, xopAL1, xopAL2, xopAM, xopAO, xopAP, xopAQ, xopAR, xopAS, xopAT, xopB, xopC1, xopD, xopE1, xopF2, xopH, xopI, xopJ1, xopJ2, xopJ3, xopJ4, xopJ5, xopO, xopP, xopT, xopU, xopW, xopY, xopZ2*	*avrBs2, avrXccA1, hpaA, hprW, xopA, xopAF, xopAH, xopAI, xopAQ, xopE2, xopE3,xopF1, xopG, xopK, xopL, xopN, xopQ, xopR, xopV, xopX, xopZ*
Type IV Secretion System	*virB1, virB2, virB3, virB4, virB5, virB6, virB7, virB8,virB9,virB10, virB11, virD4*	*tfc1, tfc7, tfc11, tfc17, tfc18, tfc20, tfc21*	*tfc2, tfc3, tfc4, tfc5, tfc6, tfc8, tfc9, tfc10, tfc12, tfc13, tfc14, tfc15, tfc16, tfc19, tfc22, tfc23, tfc24*

^a^CITA 44 did not present any unique component putatively involved in the analysed features

In the genus *Xanthomonas*, 24 structural and regulatory components of the T3SS have been determined. They are present in the *hrp* gene cluster which is regulated by the master regulons HrpG and HrpX [47]. Strain CITA 44 did not contain any of the 24 components of this gene cluster except two coding sequences which correspond to *hrpG* and *hrpX* homologs. The absence of T3SS has also been reported for another *X. arboricola* strain isolated from barley as well as for *X. cannabis* [10, 48]. The absence of the genes *hrcC*, *hrcJ*, *hrcN*, *hrcR*, *hrcS*, *hrcT*, *hrcU*, *hrcV*, *hrpB1*, *hrpD5* and *hrpF* was corroborated by conventional PCR as previously described [36]. In the case of strain IVIA 2626.1, 22 out of the 24 components, as well as homologs for the two master regulons were present, but no homologs for *hpaF* and *hrpB5* were found. Homologs for these two genes were also absent in all the genome sequences of *X. arboricola* publicly available. Sixty T3Es described in genus *Xanthomonas* were absent in strain CITA 44 and absence of 21 of them, identified in *X. arboricola* pv. pruni, was corroborated by conventional PCR using specific primers [36]. On the other hand, strain IVIA 2626.1 contained 22 T3Es, 21 of them were described previously in other *X. arboricola* pv. pruni strains [36]. In addition to these effectors, a homolog of *xopAQ* was found. Both strains contained all 12 components associated with *Agrobacterium tumefaciens* [46, 49] VirB/VirD4 T4SS [36]. Additionally, strain IVIA 2626.1 harbored a gene cluster homologous to the type four conjugation cluster (*tfc*). This cluster is composed by 24 genes associated with the expression of a conjugative pilus which is involved in the propagation of genomic islands [50]. In strain IVIA 2626.1, 17 out of the 24 genes associated with the T4SS were found and, within them, *tfc2*, *tfc4*, *tfc12*, *tfc14*, *tfc16*, *tfc22* and *tfc23* were identified as the core components required for the functioning of this T4SS [50].

An additional feature of the *X. arboricola* pv. pruni sequence is the presence of the plasmid pXap41 (41,102 Kbp) [12]. This plasmid is exclusively in *X. arboricola* pv. pruni strains and is associated with virulence because it contains some T3Es such as XopE3. Genome alignment of the plasmid pXap41 nucleotide sequence and the draft genome sequence for strain IVIA 2626.1 showed a region of 41.1 Kbp which was 99.90 % similar to the pXap41 plasmid of *X. arboricola* pv. pruni strain CFBP 5530. Conversely, no sequence region in the strain CITA 44 draft genome was similar to this plasmid. Negative results in the amplification of the genes *repA1*, *repA2* and *mobC* associated with pXap41 [12] confirmed the absence of this plasmid in strain CITA 44.

## Conclusions

Here we report and describe the draft genome sequence for two *X. arboricola* strains, CITA 44 and IVIA 2626.1, isolated from *Prunus* in Spain and associated with bacterial spot of stone fruits and almond by PCR protocols for identification of this pathovar [51, 52]. The phenotype of these two strains varied for motility and virulence. Initial genomic analysis identified several differences associated with motility (Type IV pilus) and virulence (T3SS, T3Es and T4SS), including the presence of the putative virulence plasmid pXap41 only in *X. arboricola* pv. pruni IVIA 2626.1 and the absence of the T3SS, T3Es and the plasmid pXap41 in the avirulent strain CITA 44. All these features make the avirulent strain a candidate for comparative studies to elucidate the molecular processes associated with the plant host interaction and virulence for strains of *X. arboricola* on *Prunus* species. Likewise, comparative genomic studies with related strains could provide target sequences for design of molecular diagnostics for the different pathovars of *X. arboricola*, as well as to differentiate between virulent and avirulent strains. Further functional studies will also provide insights into the pathogenesis process for *X. arboricola* strains associated with bacterial spot of stone fruits and almond.

## Abbreviations

Cv: cultivar
pv: pathovar
var: variety
